# Immune Infiltrates of m^6^A RNA Methylation-Related lncRNAs and Identification of PD-L1 in Patients With Primary Head and Neck Squamous Cell Carcinoma

**DOI:** 10.3389/fcell.2021.672248

**Published:** 2021-06-04

**Authors:** Zi-Yi Feng, Hao-Yu Gao, Tian-Da Feng

**Affiliations:** ^1^Department of Plastic Surgery, The First Hospital of China Medical University, Shenyang, China; ^2^Department of Students, The First Hospital of China Medical University, Shenyang, China; ^3^Department of Neurosurgery, Shengjing Hospital of China Medical University, Shenyang, China

**Keywords:** HNSCC, m^6^A regulators, PD-L1, lncRNA, biomarker, survival analysis, tumor microenvironment

## Abstract

**Background:** The purpose of this study was to determine the association between m^6^A-modified lncRNAs, immune infiltration, and PD-L1 expression in patients with primary head and neck squamous cell carcinoma (HNSCC) and the prognostic value of m^6^A RNA methylation-related lncRNAs in HNSCC.

**Methods:** We downloaded the RNA-seq transcriptome data and the clinical information for HNSCC from the TCGA databases and used consensus clustering analysis to divide the samples into two groups. To identify a risk signature, least absolute shrinkage and selection operator (LASSO) analyses were conducted. the association between m^6^A-modified lncRNAs, immune infiltration, and PD-L1 expression were detected by using the R packages. What is more, we used cBioPortal tools to identify genomic alterations and PD-L1 mutations and Gene set enrichment analysis (GSEA) was utilized to predict downstream access of two clusters.

**Results:** Notably, lncRNAs play significant roles in tumorigenesis and development. In total, we identified two subtypes of HNSCC according to consensus clustering of the m^6^A RNA methylation-related lncRNAs, and the T, grade and age were proven to be related to the subtypes. The Cox regression and LASSO analyses identified a risk signature including GRHL3-AS1, AL121845.4, AC116914.2, AL513190.1. The prognostic value of the risk signature was then proven. The selected gene PD-L1 mutations and the immune infiltration in both groups were further explored.

**Conclusion:** Collectively, our study elucidated the important role of m^6^A RNA methylation- related lncRNAs in tumor microenvironment of HNSCC. The proposed m^6^A RNA methylation- related lncRNAs might serve as crucial mediators of tumor microenvironment of HNSCC, representing promising therapeutic targets in improving immunotherapeutic efficacy.

## Introduction

Head and neck squamous cell carcinoma (HNSCC) is the sixth leading cause of cancer deaths in Europe and North America and comprises the majority of head and neck tumors. Five hundred thousand new HNSCC cases are diagnosed globally each year (Marur and Forastiere, 2016; Solomon et al., 2018). HNSCC can arise from the mucosal linings of several anatomical sites including the larynx, hypopharynx, nasopharynx, and oropharynx and has unpredictable, high levels of heterogeneity (Leemans et al., 2018). Although the carcinogenesis of HNSCC is not fully understood, tobacco use, excessive alcohol consumption, and human papillomavirus (HPV) infection are generally considered risk factors, especially for HNSCC arising in the oropharynx (Rassy et al., 2019). Conventional treatment for HNSCC includes surgery, chemotherapy, and radiotherapy, but these result in a low curative rate of approximately 50% and a high recurrence rate. Therefore, HNSCC is considered lethal and resistant to therapy, but there has been little improvement in HNSCC treatment over the last 50 years (Bozec et al., 2019). Nevertheless, recent genetic landscape studies have revealed a vast number of mutations that regulate squamous differentiation and act as drivers of cell malignancy, and these are potential therapeutic targets for immunotherapy (Stransky et al., 2011). Consequently, there is a tremendous need to understand the underlying molecular mechanisms of HNSCC malignancy and to explore additional novel targets for HNSCC treatment.

In recent years, there has been increased research on RNA epigenetics in various contexts, and it has been proposed that RNA modifications fine tune the chemo-structural features of infrastructural RNAs (Jonkhout et al., 2017; Traube and Carell, 2017). RNA modifications, especially methylation, are now understood to promote translation, metabolism, splicing and stability (Lan et al., 2019). Of these RNA modifications, the N6-methyladenosine (m^6^A) modification, in which the sixth nitrogen (N) atom of adenine is methylated, is the most common RNA modification in eukaryotes and has become a new area of intense research focus. m^6^A can affect almost all aspects of mRNA metabolism, including splicing, translation, stability, and it can also affect miRNA maturation at the molecular level. It has been shown that m^6^A impacts cell development, stem cell maintenance, and mitosis; these processes are important for control and regulation of circadian rhythms and fertilization, as well as tumorigenesis (Chen et al., 2019; Sun et al., 2019).

m^6^A is regulated by methylase complexes, which either “write,” “erase,” or “read” the modification (Ji et al., 2020). Methylase complexes are generally divided into three categories. “Writers” or methyltransferases, including METTL3, METTL14, and WTAP, transfer the methyl group to the sixth nitrogen of adenine. “Erasers” or demethylases, including FTO and ALKBH5, are responsible for removing the methyl group. “Readers” are specific RNA binding proteins that recognize m^6^A and lead to its downstream effects on biological processes; these “readers” include YTHDC1, YTHDC2, YTHDF1, and YTHDF2 (Dai et al., 2018). Thus, RNA methylation is a dynamic and reversible process.

In recent years, long non-coding RNAs (lncRNAs) have also become a new area of research focus due to the popularization of functional genomics research. In many cases, lncRNAs are critical regulators of gene expression and are involved in various biological functions as well as the progression of diseases including cancer (Kumar and Goyal, 2017; Peng et al., 2017). It has been shown that lncRNA expression is correlated with the degree of tumor malignancy and that dysregulation of lncRNAs is involved in HNSCC carcinogenesis (Jiang et al., 2019; Kolenda et al., 2019; Tu et al., 2020). Due to the role of m^6^A in regulating RNA stability and metabolism, it is important to understand the role of m^6^A-modified lncRNAs in HNSCC progression, and this could be useful for identifying new biomarkers and therapeutic targets for HNSCC.

The tumor microenvironment refers to the cellular environment of tumor cells and is composed of an extracellular matrix, tumor stromal cells, and soluble molecules. Immune cells including T cells, myeloid suppressor cells, and macrophages also infiltrate the tumor microenvironment. Within the tumor microenvironment, the composition of immune cells and non-tumor stromal cells is important for the diagnosis and prognosis of the tumor (Wang et al., 2018; Hinshaw and Shevde, 2019). The programmed death-ligand 1 (PD-L1) is frequently upregulated in various types of cancers. The receptor for PD-L1, PD-1, downregulates effector T cell responses, which leads to immune suppression (Zhu and Lang, 2017). Therefore, PD-1 and its ligand PD-L1 belong to the immune checkpoint pathway, and antibody-based PD-1 and PD-L1 inhibitors can lead to persistent remission for various end-stage cancers. Immune checkpoint inhibitors, including antibodies against PD-1, PD-L1 have been breakthroughs for cancer immunotherapy. It is therefore anticipated that downregulating PD-L1 expression in the tumor microenvironment may yield therapeutic effects (Wang et al., 2016). The tumor microenvironment of HNSCC is immunosuppressive, and HNSCC escapes the immune response through multiple drug resistance mechanisms. In addition, some long non coding RNAs (lncRNAs), known as immune related lncRNAs, are considered to be the regulatory factors of immune cell specific gene expression that mediate the immune process. These immune related lncRNAs may play an important role in immunotherapy resistance (Zhou et al., 2019).

**FIGURE 1:**
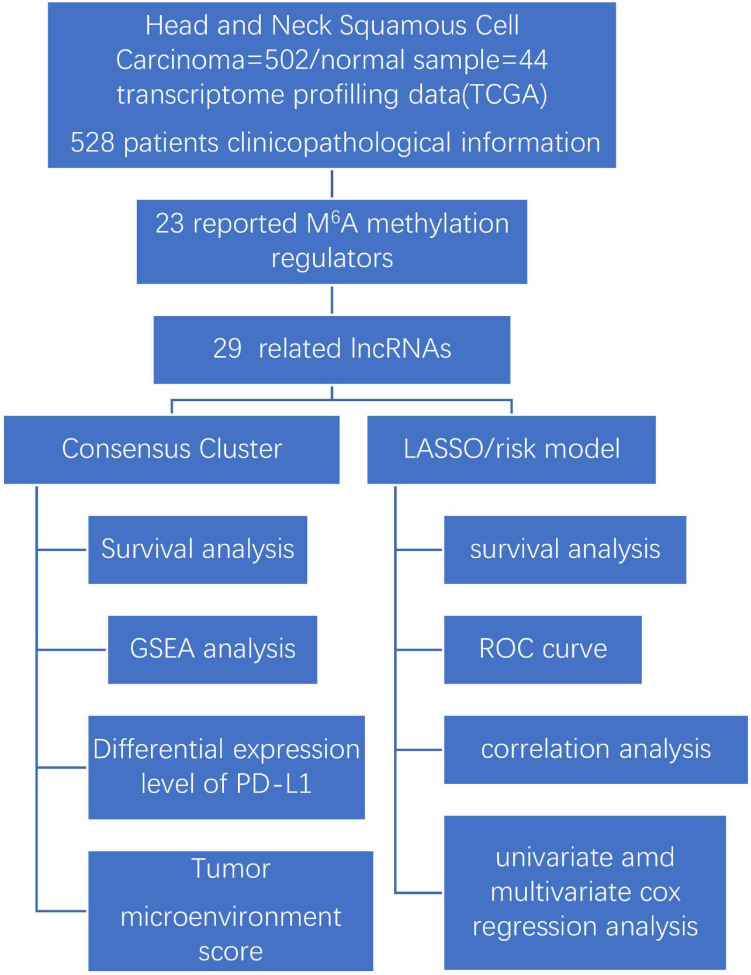
Workflow chart of data generation and analysis.

Our study explores the relationship between m^6^A-modified lncRNAs and the prognosis, PD-L1 expression, and tumor microenvironment of HNSCC. We also divided our patient cohort into two clusters and established a signature based on m^6^A-modified lncRNAs to improve prognostic risk stratification and treatment decisions in HNSCC patients. We fully analyzed the relationship between clustering subgroups, risk models, PD-L1 expression, immune scores, and immune cell infiltration. The present research also explores potential regulatory mechanisms affecting the tumor microenvironment and HNSCC immunotherapy strategies (Figure 1).

## Materials and Methods

### Data Acquisition

The following data was obtained from The Cancer Genome Atlas (TCGA) database^1^ on February 10, 2021: RNA-seq transcriptome from 502 HNSCC patients and corresponding clinicopathological data from 528 HNSCC patients; RNA-seq transcriptome from 44 healthy controls. For the clinical information downloaded from TCGA including all HNSCC patients, so it has a different number of the number of RNA-seq transcriptome of HNSCC patients and we will match them later. The RNA-seq transcriptome data were normalized by fragment per kilobase of exon model per million (FPKM, mean fragment per kilobase million). The patients’ clinicopathological information included survival state, survival time age, gender, Tumor Node Metastasis (TNM) staging, and grade. The 528 HNSCC patients were randomly divided into a train cohort and a test cohort in a 1:1 ratio using the “caret” R package^2^.

### Detection of Regulators of m^6^A RNA Methylation and Co-expression lncRNAs

To make our study more comprehensive, we selected 23 genes (METTL3, METTL14, METTL16, WTAP, VIRMA, ZC3H13, RBM15, RBM15B, YTHDC1, YTHDC2, YTHDF1, YTHDF2, YTHDF3, HNRNPC, FMR1, LRPPRC, HNRNPA2B1, IGFBP1, IGFBP2, IGFBP3, RBMX, FTO, ALKBH5) as classical regulators of m^6^A RNA methylation based on previously published studies (Tang et al., 2020; Tian et al., 2020; He and He, 2021). Expression of the 23 m^6^A regulators was then extracted from the RNA-seq transcriptome data. Co-expression analysis was conducted with the “limma” R package^3^. We then performed coexpression analysis, and the following parameters were used as filter conditions to select regulators of m^6^A lncRNA methylation: “correlation coefficient = 0.4” and “pvalueFilter = 0.001.” By using the “igraph” R package^4^, the expression data co-expression network for lncRNAs were also obtained. To observe the differences in m^6^A RNA methylation regulators and their co-expressed lncRNAs between HNSCC and control groups more clearly, heatmap and boxplot were made by using the “limma” package, and univariate Cox regression was performed to screen the signature in 29 m^6^A RNA methylation-related lncRNAs whereas a hazard ratio greater than 1 suggests an increased risk, and a hazard ratio below 1 suggests a smaller risk.

### Consensus Clustering Analysis

To understand the biological characteristics of the m^6^A-modified lncRNAs in the HNSCC cohort, we used the “ConsensusClusterPlus” package (1,000 iterations and resample rate of 80%)^5^ to assign the patients into two categories. The algorithm of random sampling was 1,000 permutations. A Kaplan–Meier analysis was then conducted to determine the overall survival (OS) of the two clusters. A heatmap was generated to depict the relationship between grouping and clinicopathological factors, using the “pheatmap” R package^6^. Gene set enrichment analysis (GSEA) 4.1.0 was utilized to predict the potential functions and downstream access of the two clusters.

### Construction of the Prognostic Signature

Using the least absolute shrinkage and selection operator (LASSO) regression analysis we established prognostic risk signatures of m^6^A-modified lncRNAs. The coefficients were obtained from the LASSO regression algorithm, and the risk score was calculated by the following formula (Huang et al., 2020):

(1) $$\mathrm{Riskscore}=\sum_{i=1}^{n}\mathrm{codfi}{}^{*}x_{i}$$

where Codfi is the coefficient and x_*i*_ is the transformed relative expression value of each selected lncRNA. Using this method, the risk score of each patient in the train and test groups was calculated. Samples in the train- and test- groups were divided into high- and low-risk groups with the median risk score used as the cutoff point.

### Evaluating the Prognostic Value of the lncRNAs Signature

Kaplan–Meier analysis was conducted to assess the overall survival difference between the high- and low-risk groups in the train and test group. To analyze the predictive efficacy of the signatures, receiver operating characteristic (ROC) curves were implemented, and the area under the curve (AUC) was calculated. The distribution of clinicopathological features in high- and low risk groups were visualized by “pheatmap” R package with heatmaps. For univariate and multivariate analyses, Cox regression models were used to evaluate whether risk scores would serve as independent prognostic factors when integrated with other clinical features.

### Genomic Alteration and Co-expression Level of PD-L1

The mutations and putative copy number alterations of PD-L1 in HNSCC were extracted from the cBioPortal tool^7^. The OncoPrint dsiplayed the overview of genetic alterations of PD-L1 in HNSCC samples. The “limma” package was used to visualize the expression differences of PD-L1 between the two patient clusters, normal and tumor samples as well as the high- and low-risk groups. The package “corrplot” was used to depict the association between PD-L1 expression and m^6^A-modified lncRNAs.

### Evaluation of Immune Infiltration

The “estimate”^8^ R package (Yoshihara et al., 2013) was used to calculate immune-scores in the HNSCC patients using the ESTIMATE algorithm. What is more, to obtain the fraction scores for 22 immune cell subtypes in each tumor sample, we performed cell type identification by estimating relative subtypes of RNA transcripts (CIBERSORT)^9^. Here, the 1,000 permutations algorithm was employed, and only samples with P less than 0.05 were considered for further analysis. Differential immune infiltration levels between the subgroups were then compared by clustering and risk scores.

### Statistical Analysis

R software (Version 4.0.3) was used for all statistical analyses, and the data are shown as mean ± standard deviation. Differences between the two groups and among multiple groups were analyzed using the default Wilcoxon test and one-way analysis of variance (ANOVA), respectively. The differences in overall survival between groups were determined via Kaplan–Meier analysis and a log-rank test. The subtypes, clinicopathological features, risk scores, PD-L1 expression, and immune infiltration levels were determined by a Pearson correlation test. Results were considered statistically significant when the P was less than 0.05. In some cases, “*P* < 0.05” gave too many results, and in these scenarios, we used “*P* < 0.01” as the filter factor. We will explain it in the result presentation section later.

## Results

### Differential Expression of m^6^A RNA Methylation Regulators and m^6^A-Modified lncRNAs in Head and Neck Squamous Cell Carcinoma and Normal Tissue

m^6^A RNA methylation regulators are necessary for the initiation and progression of cancer (Sun et al., 2019). To assess the biological role of m^6^A RNA methylation regulators in HNSCC, we studied the expression of 23 m^6^A regulatory genes in HNSCC and adjacent normal tissue using RNA-seq data systematically downloaded from The Cancer Genome Atlas (TCGA). Our analysis included 502 tumor samples and 44 healthy tissue controls.

**FIGURE 2:**
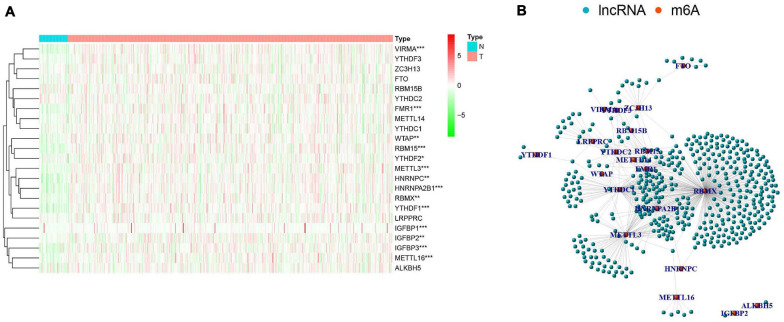
The expression characteristics and correlations of N6-methyladenosine (m^6^A) RNA methylation regulators in head and neck squamous cell carcinoma (HNSCC). **(A)** Heatmap presented the overall expression of 23 m^6^A RNA methylation regulators in HNSCC tissues (T) and normal tissues (N) from The Cancer Genome Atlas (TCGA) datasets. *P* < 0.05 (”*”), *P* < 0.01 (”**”), and *P* < 0.001 (”***”). **(B)** The interaction of the m^6^A RNA methylation regulators (red) and related IncRNAs (blue).

There were clear differences in the expression of m^6^A RNA methylation regulators between HNSCC samples and healthy controls. From our heatmap (Figure 2A), we observed that “readers” (IGFBP3, IGFBP1, HNRNPA2B1, YTHDF1, FMR1, HNRNPC, RBMX) and “writers” (VIRMA, METTL3, METTL16, RBM15, WTAP) were upregulated in the HNSCC samples, compared to the controls. There was no statistically significant difference between tumor and normal tissue in the expression of “readers” (IGFBP2, YTHDF2, YTHDC2, YTHDF3, LRPPRC, YTHDC1), “writers” (METTL14, ZC3H13, RBM15B) and “erasers” (FTO, ALKBH5). These findings demonstrate that m^6^A RNA methylation regulators play a significant role in HNSCC. To further investigate the connections between the 23 m^6^A RNA methylation regulators and related lncRNAs, we determined the lncRNAs co-expressed with the 23 m^6^A RNA methylation regulators by analyzing RNA-seq transcriptome data and visualizing using a co-expression network (Figure 2B). The red nodes represent the m^6^A RNA methylation regulators, and the blue nodes are lncRNAs. We observed a close relationship between m^6^A methylation regulators and m^6^A-modified lncRNAs in which RBMX, HNRNPA2B1and FMR1 are considered as hub genes.

**FIGURE 3:**
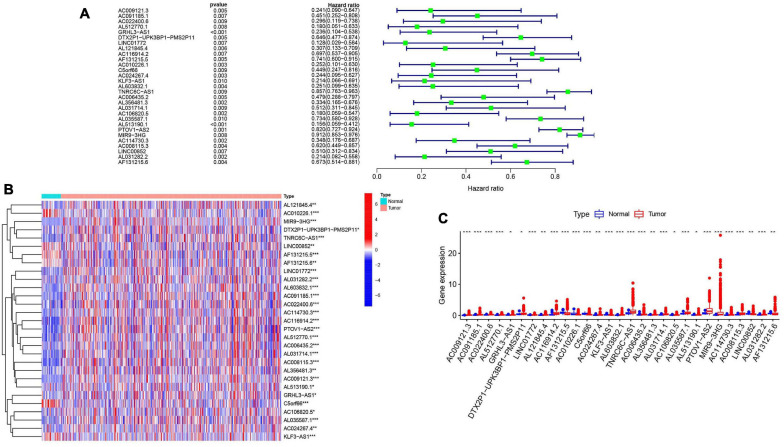
m^6^A RNA methylation-related IncRNAs regulators in HNSCC in TCGA cohort. **(A)** Univariate Cox regression was performed to screen the signature in 29 m^6^A RNA methylation-related IncRNAs. **(B)** Heatmap presented the overall expression of 29 m^6^A RNA methylation-related IncRNAs in HNSCC tissues and normal tissues from The Cancer Genome Atlas (TCGA) datasets. *P* < 0.05 (”*”), *P* < 0.01 (”**”), and *P* < 0.001 (”***”). **(C)** The differential expression of the m^6^A RNA methylation-related IncRNAs was visualized by boxplot (blue means normal tissues; red means HNSCC samples).

To further explore the relationship between m^6^A-modified lncRNAs and HNSCC prognosis, we obtained the relevant clinical data from TCGA and merged the data on survival time, survival status, and expression of m^6^A-modified lncRNAs. Using the R package “survival” with the screening condition “*P* < 0.05,” we selected 29 lncRNAs which were closely related to the prognosis of HNSCC. We performed univariate Cox analyses (Figure 3A) to analyze the relationship between these 29 lncRNAs and overall survival and found that all 29 lncRNAs were protective, and their expression was highly correlated with a positive prognosis. We then comprehensively investigated expression differences of the 29 m^6^A-modified lncRNAs between HNSCC patients and healthy controls in the TGCA dataset. We visualized this data using heatmap (Figure 3B) and box plots (Figure 3C) and found that the expression of these lncRNAs differed significantly between HNSCC patients and healthy controls, most lncRNAs are highly expressed in tumor group, except AL121845.4, LINC00852, AF131215.5, AF131215.6, and C5orf66.

### Significant Correlation Between Consensus Clustering of m^6^A-modified lncRNAs and Characteristics and Survival of HNSCC Patients

**FIGURE 4:**
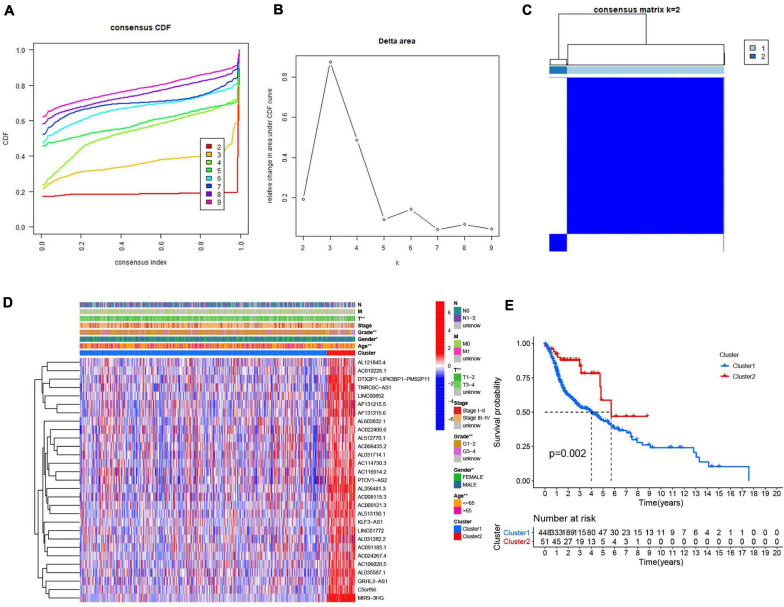
Association between the m^6^A RNA methylation-related IncRNAs and clinicopathological and prognostic features of the HNSCC patients. **(A)** Consensus clustering model with cumulative distribution function (CDF) for *k* = 2–9 (k means cluster count). **(B)** Relative change in area under the CDF curve for *k* = 2–9. **(C)** The Cancer Genome Atlas (TCGA) HNSCC cohort was classified into two clusters with *k* = 2. **(D)** The correlation of the two clusters with clinicopathologic features was visualized by heatmap. *P* < 0.05 (”*”), *P* < 0.01 (”**”). **(E)** The overall survival of HNSCC patients in the two clusters was calculated by Kaplan-Meier curves.

Consensus clustering analysis was conducted, with *k* = 2–9 in a cumulative distribution function (CDF) (Figures 4A,B), k means the cluster count. Depending on the similarity of the expression of m^6^A-modified lncRNAs and the proportion of ambiguous clustering measures, *k* = 2 (Figure 4C) was determined to be the optimal clustering parameter (Supplementary Figure 1). We combined the survival time of patients and the expression level of the selected lncRNAs, and the incomplete samples were removed. Finally, 499 patients were obtained and divided into two clusters, cluster 1 (*n* = 448) and cluster 2 (*n* = 51), based on expression of the m^6^A-modified lncRNAs. Patients in cluster 2 had higher m^6^A-modified lncRNA expression levels than patients in cluster 1. We then compared the clinicopathological features of the two clusters and their correlation were tested, then we found that the tumor size, grade, age, and gender were closely associated with our cluster analysis (Figure 4D). Overall survival (Figure 4E) was higher in cluster 2 (OS, *p* = 0.002) than in cluster 1.

**FIGURE 5:**
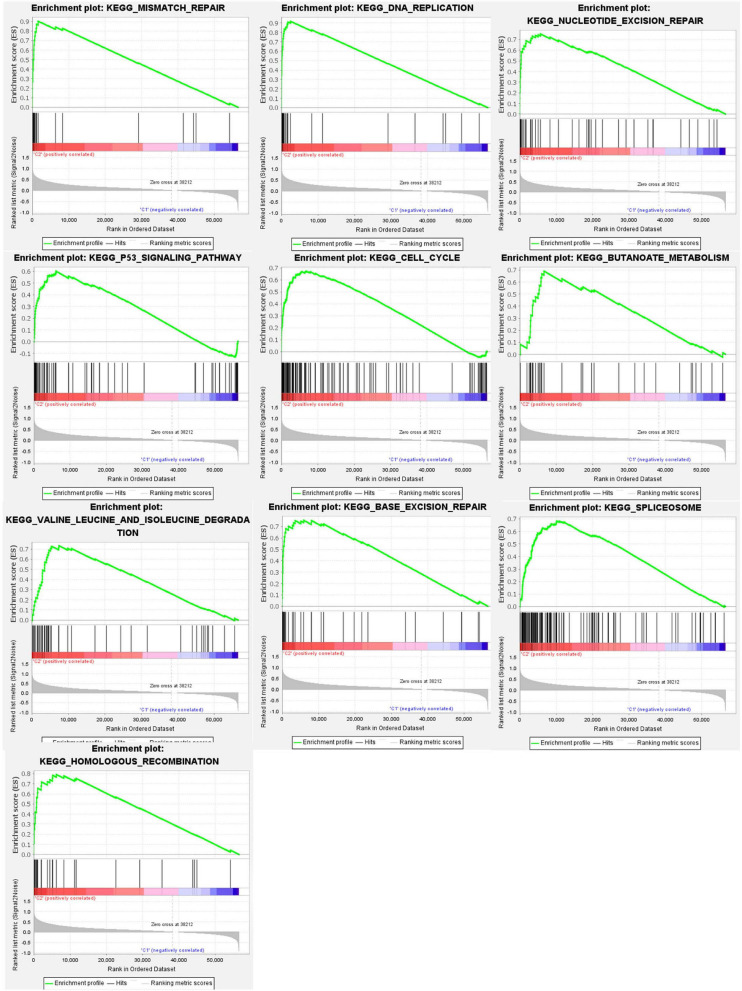
Gene set enrichment analysis (GSEA) was conducted to predict the potential functions and pathways between the two clusters.

Gene set enrichment analysis (GSEA) (Figure 5) revealed active pathways that varied between the two clusters. Using false discovery rate (FDR) *q-*value < 0.01 as the filter condition, we found that the following pathways were active in cluster 2: “mismatch repair,” “DNA replication,” “nucleotide excision repair,” “P53 signaling pathway,” “cell cycle,” “butanoate metabolism,” “valine leucine and isoleucine degradation,” “base excision repair,” “spliceosome,” “homologous recombination.” However, using the same filter conditions, we did not find active pathways in cluster 1. This absence of active pathways in cluster 1 could indicate that our algorithm is not as sensitive as we had anticipated or that a larger sample size is needed.

### Relationship Between PD-L1 Expression and m^6^A-Modified lncRNAs

**FIGURE 6:**
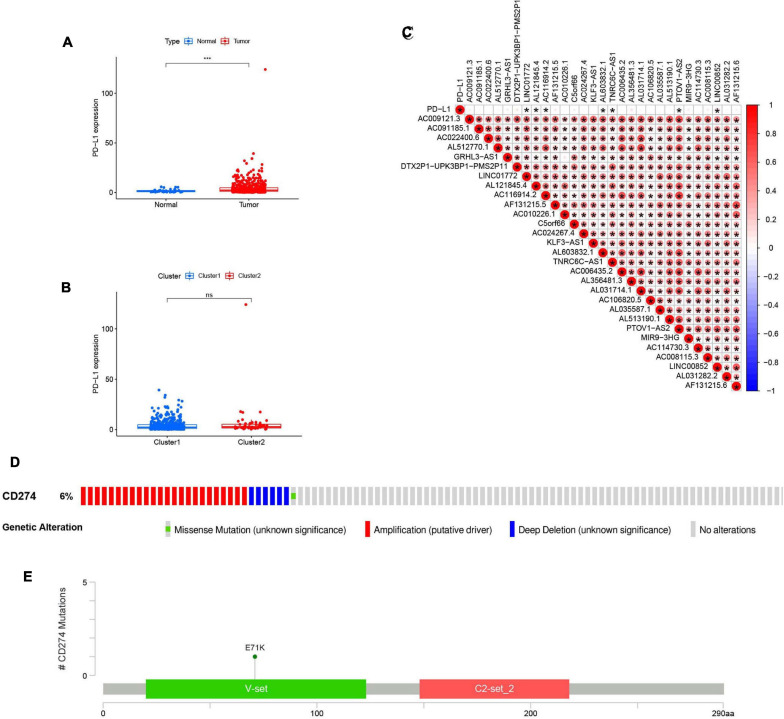
Association of PD-L1 with m^6^A RNA methylation-related IncRNAs. **(A)** PD-L1 upregulation in HNSCC in TCGA cohort, *P* < 0.001 (“***”). **(B)** The expression level of PD-L1 in clusterl/2 subtypes in TCGA cohort. **(C)** The expression correlation of the m^6^A RNA methylati on-related IncRNAs and PD-L1, the red circle represents a positive correlation. *P* < 0.05 (“*”). **(D)** OncoPrint of PD-L1 alterations in HNSCC cohort identified by cBioPortal. **(E)** Lollipop of PD-L1 alterations in HNSCC cohort identified.

To determine the relationship between PD-L1 and m^6^A-modified lncRNAs, we estimated the difference in PD-L1 expression between tumor samples and healthy controls (Figure 6A) and between clusters 1 and 2 (Figure 6B). Compared to normal adjacent tissues, PD-L1 expression was upregulated in tumor samples (*p* < 0.001). However, there was no statistically significant difference in PD-L1 expression between clusters 1 and 2. Expression of PD-L1 was associated with several lncRNAs, including LINC01772, AL121845.4, AC116914.2, AL603832.1, TNRC6C-AS1, PTOV1-AS2, LINC00852. Furthermore, the 29 lncRNAs were positively correlated with each other (Figure 6C).

cBioPortal was used to determine the types and frequency of PD-L1 mutations in HNSCC. According to Oncoprint (Figure 6D), PD-L1 is altered in six percent of HNSCC patients, and these alterations include missense mutations, amplifications, and deep deletions. The majority of PD-L1 alterations in HNSCC are amplifications. A lollipop diagram (Figure 6E) of PD-L1 was generated to show the locations of PD-L1 mutations in HNSCC patients including the V-set domain. We also compared the overall survival (Supplementary Figure 2A) and the disease-free survival (Supplementary Figure 2B) between patients with and without PD-L1 mutations and found that there were no statistically significant differences.

### Consensus Clustering for m^6^A-Modified lncRNAs Associated With Distinct Immune Cell Infiltration and Tumor Microenvironment Differences

**FIGURE 7:**
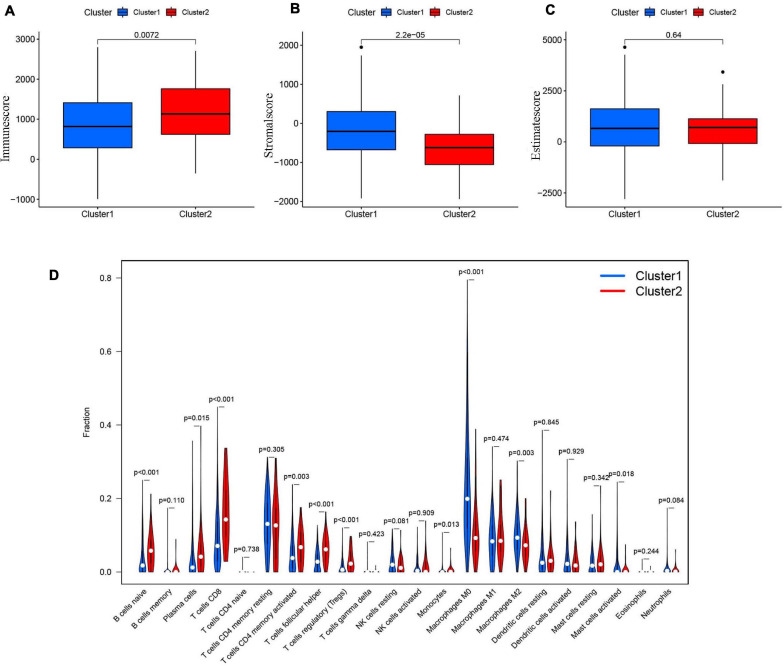
**(A)** The Immunescore in cluster 1/2 subtypes in TCGA cohort. **(B)** The Stromal-score in cluster 1/2 subtypes in TCGA cohort. **(C)** The Estimatescore in cluster 1/2 subtypes in TCGA cohort. **(D)** The infiltrating levels of 22 immune cell types in clusterl/2 subtypes in the TCGA cohort.

The tumor and its environment are simultaneously interdependent and antagonistic with one another. This is a key and core challenge in modern tumor biology. In recent years, with advances in tumor cytology and molecular biology, there has been a greater understanding of the relationship between the tumor and its environment. Not only is this relationship important for understanding mechanisms of tumor occurrence, development, and metastasis, it could also be valuable for cancer diagnosis, and prognosis. Two of the main non-tumor components of the tumor microenvironment are immune cells and stromal cells (Wu and Dai, 2017; Arneth, 2019). We scored the immune cells (Figure 7A) and stromal cells (Figure 7B) in each sample and added the two scores together to obtain the total estimatescore (Figure 7C). Higher total scores indicated lower tumor purity. There was a clear difference in the immune-scores and stromal-scores of our two clusters; immune-scores were higher in cluster 2, whereas stromal-scores were higher in cluster 1. There was not a statistically significant difference in total estimate-scores between the two clusters.

We then analyzed the proportions of 22 immune cell subtypes between clusters 1 and 2 (Figure 7D). The screening condition was a *p* < 0.001. Cluster 1 had higher infiltration of M0 macrophages, whereas cluster 2 was more closely associated with CD8 T cells, naïve B cells, regulatory T cells (Tregs), and follicular helper T cells.

### Construction and Validation of Prognostic Signatures for m^6^A-Modified lncRNAs

**FIGURE 8:**
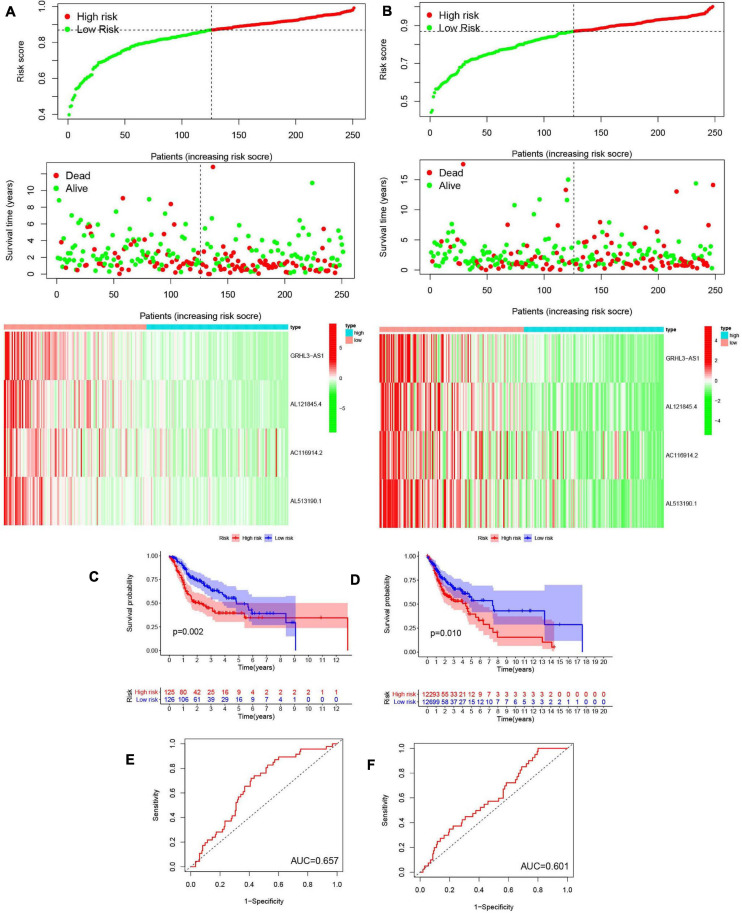
Construction and validation of prognostic signatures of m^6^A methylation regulators. **(A,B)** Distribution of risk score, OS, and OS status and heatmap of the four prognostic signatures in the train cohort **(A)** and test cohort **(B)**. **(C,D)** Kaplan-Meier curves of OS for patients with HNSCC based on the risk score in the train cohort **(C)** and test cohort **(D)**. **(E,F)** ROC curves measuring the predictive value of the risk score in the train cohort **(E)** and test cohort **(F)**.

We next evaluated the usefulness of m^6^A-modified lncRNAs for predicting patient prognosis. The 499 patients were evenly divided into two cohorts: the TCGA train cohort (251 patients) and the test cohort (248 patients). A least absolute shrinkage and selection operator (LASSO) regression analysis was conducted according to the expression levels of the 29 m^6^A-modified lncRNAs in the TCGA train cohort (Supplementary Figures 4A,B). From this, four important m^6^A-modified lncRNAs were identified, which included GRHL3-AS1, AL121845.4, AC116914.2, AL513190.1. The risk scores of the train and the test cohorts were estimated using the coefficients from the LASSO algorithm. The formula was as follows: risk score = − (0.414908709551883 ^∗^ GRHL3-AS1 expression level+ 0.326529379046119 ^∗^ AL121845.4 expression level+ 0.0128315743810079 ^∗^ AC116914.2 expression level+ 0.260494245733385 ^∗^ AL513190.1 expression level). Patients in the HCSCC train and test groups were then split into high- and low-risk groups based on their median risk scores. The relationships between risk score, OS, OS status, and expression signatures of the four m^6^A-modified lncRNAs in the train and test cohorts are shown (Figures 8A,B), The heatmap results indicated that the four selected lncRNAs were highly expressed in the low-risk group.

Meanwhile, we further analyzed OS between the two groups (Figures 8C,D) and found that OS was significantly greater in the low-risk group than in the high-risk group, regardless of whether patients were in the train (*p* = 0.002) or test group (*p* = 0.010). To further explore the sensitivity and specificity of the risk signatures for diagnosis, a ROC curve was applied and the AUC values for the risk signatures were 0.657 and 0.601, respectively, in the train and the test cohorts (Figures 8E,F).

Thus, the risk signature score could somewhat predict survival rates of HNSCC patients, and it could discriminate patient prognosis remarkably well.

### Prognostic Risk Scores Correlated With Clinicopathological Factors, Clusters, and Immune-Scores in HNSCC

**FIGURE 9:**
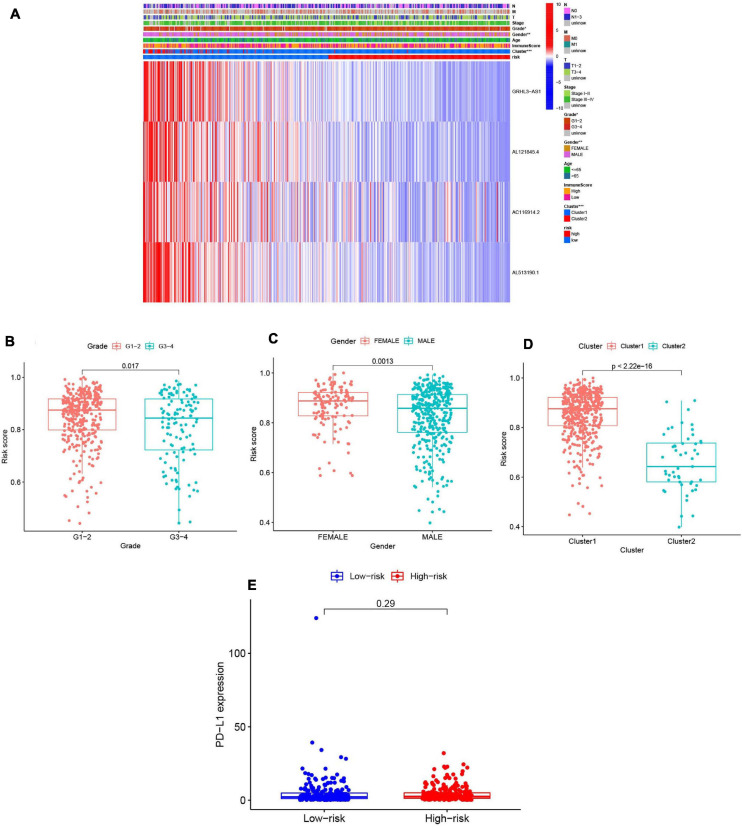
Prognostic risk scores correlated with clinicopathological features and immunoscore in TCGA training cohort. **(A)** Heatmap and clinicopathologic features of high- and low-risk groups. *P* < 0.05 (”*”), *P* < 0.01 (”**”), and *P* < 0.001 (”***”). **(B–D)** Distribution of risk scores stratified by grade **(B)**, gender **(C)**, and clusterl/2 **(D)**. **(E)** The PD-Ll expression level by risk score group in TCGA training set.

We summarized information from all of the samples in the high- and low-risk groups from the train and test groups and compared their clinicopathological factors, cluster analysis results, and the immune-scores. Our heatmap (Figure 9A) also visualized expression differences of the four selected m^6^A-modified lncRNAs between the high- and low-risk groups. Absolute expression of the four m^6^A-modified lncRNAs was lower in the high-risk group than in the low-risk group, which indicates that they are protective. Grade 1–2 (Figure 9B), female (Figure 9C) and cluster 1 (Figure 9D) showed higher risk scores. We then further evaluated the PD-L1 expression and risk score, however, no significant correlation was found (Figure 9E).

To verify the utility of our model for use in different clinical groups, the differences in OS of the high- and low-risk groups among age, gender, grade, stage, Tumor Node Metastasis (TNM) staging were all determined. We observed that except in female group and the grade 1–2 group, in the other groups, all the rest OS in low-risk groups were higher than high-risk group (Supplementary Figure 5). This further proved that our model was meaningful.

**FIGURE 10:**
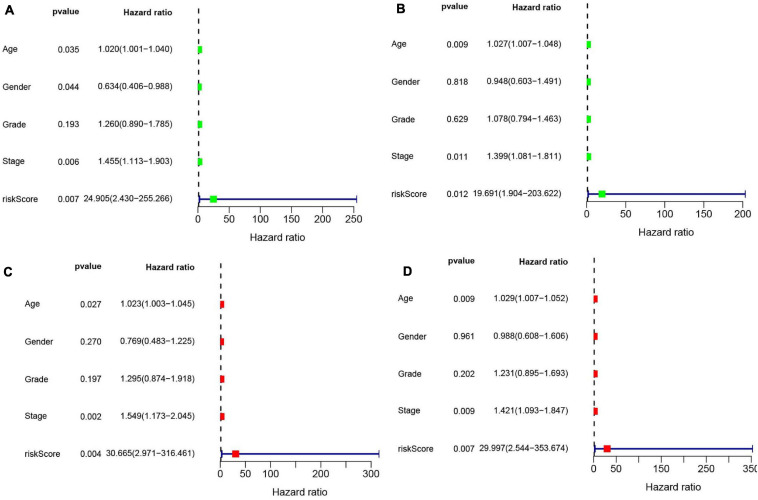
**(A)** Univariate Cox regression was performed in the train group. **(B)** Univariate Cox regression was performed in the test group. **(C)** Multiple Cox regression was performed in the train group. **(D)** Multiple Cox regression was performed in the test group.

Univariate (Figures 10A,B) and multivariate Cox (Figures 10C,D) analyses for OS in the train and test groups were performed to determine whether clinicopathological characteristics (including age, gender, grade, stage, and risk score) were independent prognostic factors. The Cox proportional hazards model was applied for all variables and was used in the univariate analysis. Our findings showed that age, gender, stage, and risk score were independent factors for poor prognosis of patients in the train group, and age, stage, and risk score were independent factors in the test group. Multivariate analysis using the same variables as the univariate analysis further indicated that age, stage, and risk score were independent factors for poor prognosis in both the train and test groups. The present results indicate that the risk score has remarkable value for predicting patient prognosis.

### Correlation of m^6^A-Modified lncRNAs With Immunocytes

**FIGURE 11:**
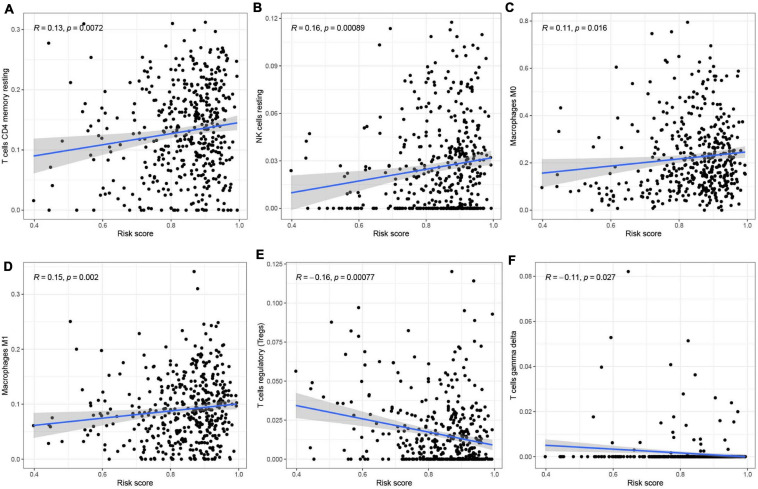
Relationships between the risk score and infiltration abundances of six immune cell types. **(A–F)** T cells CD4 memory resting **(A)**, NK cells resting **(B)**, Macrophages MO **(C)**, Macrophages Ml **(D)**, T cells regulatory (Tregs) **(E)**, and T cells gamma delta **(F)**.

To analyze the effect of the four m^6^A-modified lncRNAs on the HNSCC immune microenvironment, we correlated risk score with the infiltration of ten immune cell subtypes. There was a significant positive correlation between risk score and populations of CD4 memory resting T cells (*p* < 0.01) (Figure 11A), resting NK cells (*p* < 0.001) (Figure 11B), M0 macrophages (*p* < 0.05) (Figure 11C) and M1 macrophages (*p* < 0.01) (Figure 11D). The risk score was negatively correlated with infiltration of Tregs (*p* < 0.001) (Figure 11E) and gamma delta T cells (*p* < 0.05) (Figure 11F). This finding confirms that m^6^A-modified lncRNAs -based risk signature can be implicated in the immune microenvironment of HNSCC, so as to promote individual treatment strategies and expand insights to accelerate the advancement of therapeutic approaches.

## Discussion

In recent years, genome-wide research has shown that most genes are transcribed and form an RNA network in cells comprised of large and small RNAs (Pandiani et al., 2021). However, only a small part of these RNAs can be translated into proteins. During translation process, the RNA modification after transcription is a critical step, and approximately 150 post-transcriptional RNA modifications have been demonstrated across species (Motorin and Helm, 2011; Ogawa et al., 2021). Among these modifications, N6-methyladenosine (m^6^A) is the most common among eukaryotic mRNAs and long non-coding RNAs. m^6^A can determine whether mRNAs are translated or undergo decay, and this can lead to differences in cell differentiation, embryonic development, and stress responses (Zhao et al., 2017). Long non-coding RNAs (lncRNAs) are non-coding transcripts that are usually longer than 200 nucleotides, and they are one of the largest and most diverse categories of RNA. lncRNAs play vital roles in a plethora of cellular functions, and most of these require interactions with one or more RNA-binding proteins (RBPs), competing endogenous RNAs (ceRNAs) or other RNAs. Previous studies have shown that m^6^A-methylated lncRNA can significantly affect the functions of target genes in a variety of tumors through RNA-protein interactions (Ferrè et al., 2016; Paraskevopoulou and Hatzigeorgiou, 2016; Tu et al., 2020). However, it remains unclear how m^6^A-modified lncRNAs affect the expression of target genes in HNSCC. Furthermore, different cancer microenvironments are formed at each stage of cancer progression, and these microenvironments have different properties and can be both detrimental and beneficial for tumorigenesis (Kim and Bae, 2016). Therefore, there is need for a greater mechanistic understanding of how m^6^A-modified lncRNAs affect the tumor microenvironment.

The present study analyzed the effects of m^6^A-modified lncRNAs on the tumor microenvironment of HNSCC. We investigated 23 previously reported m^6^A RNA methylation regulators in TCGA HNSCC datasets. Consistently, most of the 23 m^6^A regulators were upregulated in HNSCC samples compared to normal samples. By analyzing the gene expression files, we determined the lncRNAs associated with the 23 m^6^A RNA methylation regulators and constructed a co-expression network. Univariate Cox analyses indicated that 29 lncRNAs were potential prognostic factors for HNSCC and that high expression of these lncRNAs indicated a positive tumor prognosis.

According to expression of the m^6^A-modified lncRNAs, the HNSCC cohort was spilt into two clusters using consensus clustering. Cluster 2, which had elevated expression of the 29 m^6^A-modified lncRNAs, had significantly higher survival and lower tumor stage compared to cluster 1. Furthermore, GSEA was used to analyze differential gene expression and found differences in tumor-related pathways, cell cycle, and the PI3K–AKT signaling pathway (Porta et al., 2014). These data demonstrate the underlying relationship between m^6^A-modified lncRNAs and the initiation and progression of HNSCC.

Programmed death-ligand 1 (PD-L1) is often upregulated in various cancers (Sun et al., 2018). The differences in the expression of PD-L1 in HNSCC and normal tissues and between the two clusters were detected. In the tumor group, PD-L1 expression was significantly increased compared to normal tissue, but no significant difference in two clusters. The PD-L1 mutations were also checked in HNSCC. We scored the immune cells and stromal cells in each sample found that immune-scores were significantly higher in cluster 2 whereas stromal-scores were significantly higher in cluster 1. Next, the content of 22 immune cell subtypes between clusters 1 and 2 were analyzed. Cluster 1 had greater infiltration of M0 macrophages, whereas cluster 2 had more relative CD8 T cells, Tregs, follicular helper T cells, and naïve B cells.

We next evaluated the prognostic value of the m^6^A-modified lncRNA signatures in HNSCC patients. The LASSO algorithm was applied and four lncRNAs were chosen to determine the risk signature. According to our formula, we calculated the risk value of each sample and stratified the patients into high- and low-risk groups. The OS of the low-risk group was significantly higher than that of the high-risk group, regardless of whether patients were in the train or test cohort. In the train and the test cohorts, the AUC values were 0.657 and 0.601, respectively, which indicates that the signature risk score could predict survival rates for HNSCC patients to some extent. To estimate the independent prognostic factor, univariate and multivariate Cox analyses for OS were conducted in the train and test groups. Correlation of m^6^A-modified lncRNAs with infiltration of various immunocytes confirmed that the signatures could predict the HNSCC immune microenvironment.

In summary, the present research systematically evaluated the prognostic value, the correlation with PD-L1, role in the tumor environment, and potential regulatory mechanisms of m^6^A-modified lncRNAs in HNSCC. Thus, better understanding the role of m^6^A-modified lncRNAs in the tumor microenvironment can potentially improve precision immunotherapy for HNSCC.

## Data Availability Statement

Publicly available datasets were analyzed in this study. This data can be found here: http://cancergenome.nih.gov.

## Author Contributions

H-YG and Z-YF: conceptualization. T-DF: original manuscript preparation. Z-YF: draft correction, supervision and editing. All authors listed have made substantial contribution to the manuscript, which is acknowledged and confirmed by themselves, read and agreed on the final version of the manuscript.

## Conflict of Interest

The authors declare that the research was conducted in the absence of any commercial or financial relationships that could be construed as a potential conflict of interest.

## Footnotes

1 http://cancergenome.nih.gov/

2 https://CRAN.R-project.org/package=corrplot

3 http://www.bioconductor.org/packages/release/bioc/html/limma.html

4 https://CRAN.R-project.org/package=igraphdata

5 https://bioconductor.org/packages/ConsensusClusterPlus/

6 https://CRAN.R-project.org/package=pheatmap

7 http://cbioportal.org

8 https://bioinformatics.mdanderson.org/estimate/rpackage.html

9 https://cibersort.stanford.edu/
