# Supplementary figures and images for: Immune Infiltrates of m6A RNA Methylation-Related lncRNAs and Identification of PD-L1 in Patients With Primary Head and Neck Squamous Cell Carcinoma
Source: Front Cell Dev Biol. 2021 Jun 4;9:672248. doi: 10.3389/fcell.2021.672248 (PMC8220827; doi:10.3389/fcell.2021.672248)

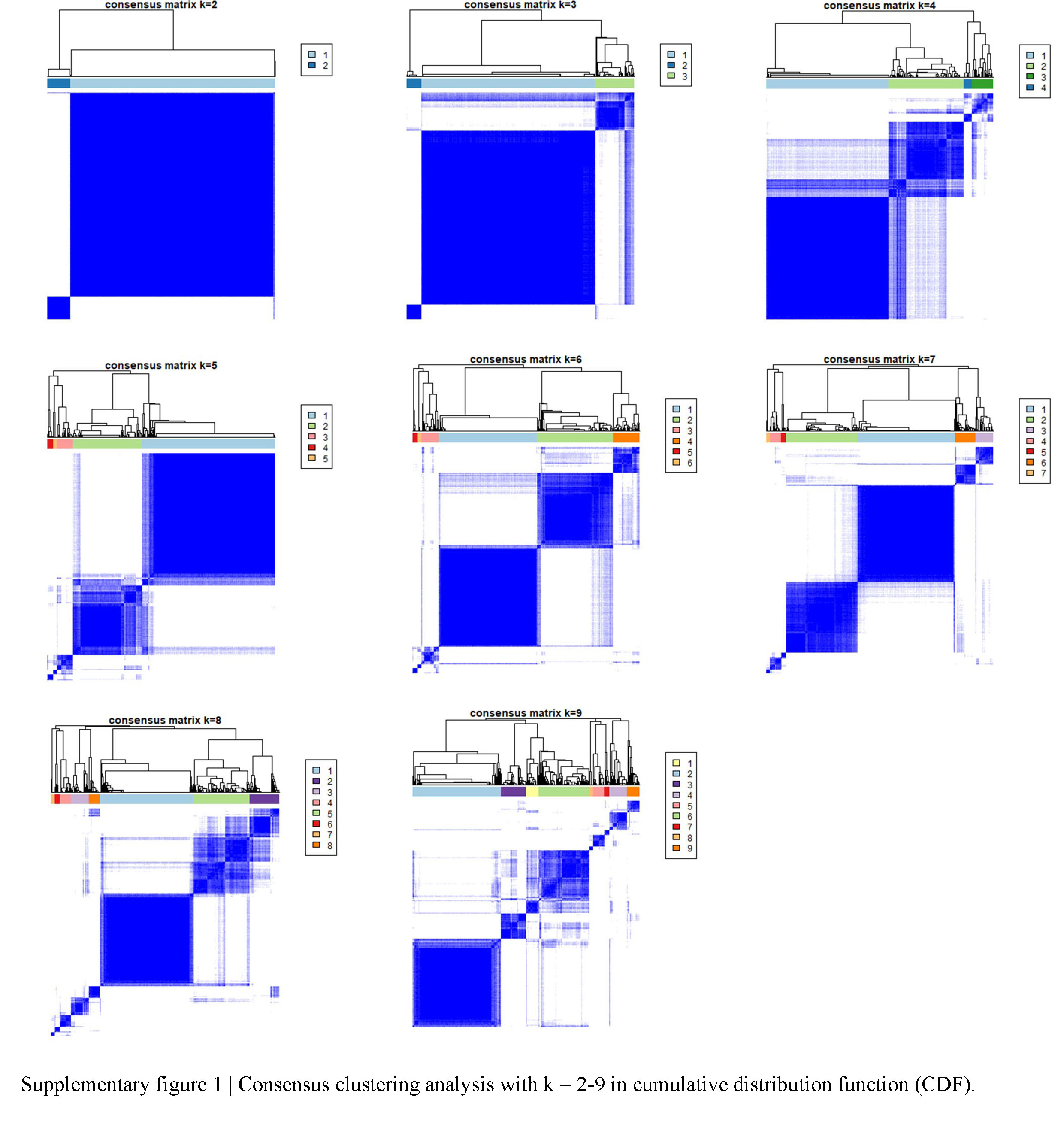

Supplement: Supplementary file 1 [file Image_1.JPEG]

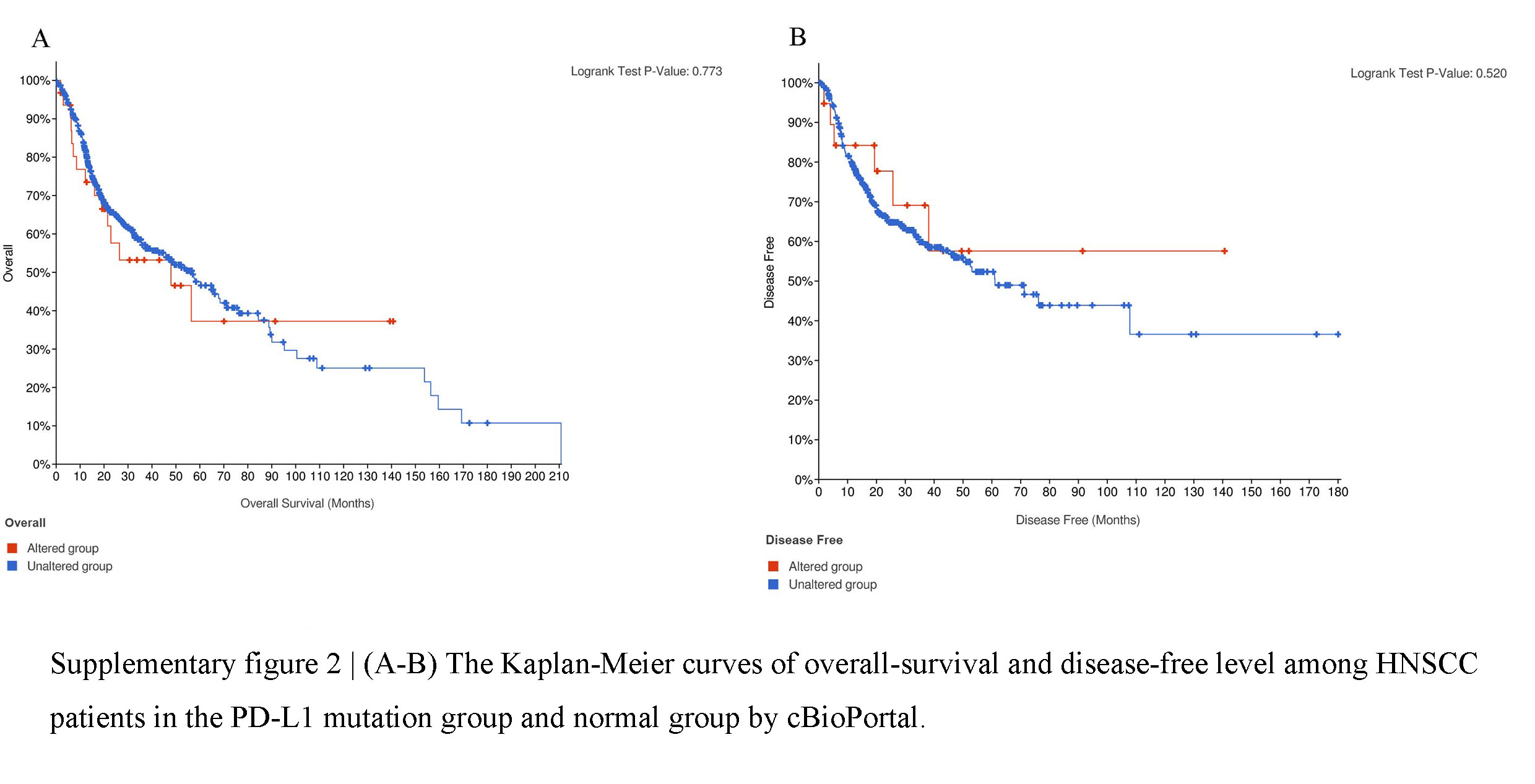

Supplement: Supplementary file 2 [file Image_2.JPEG]

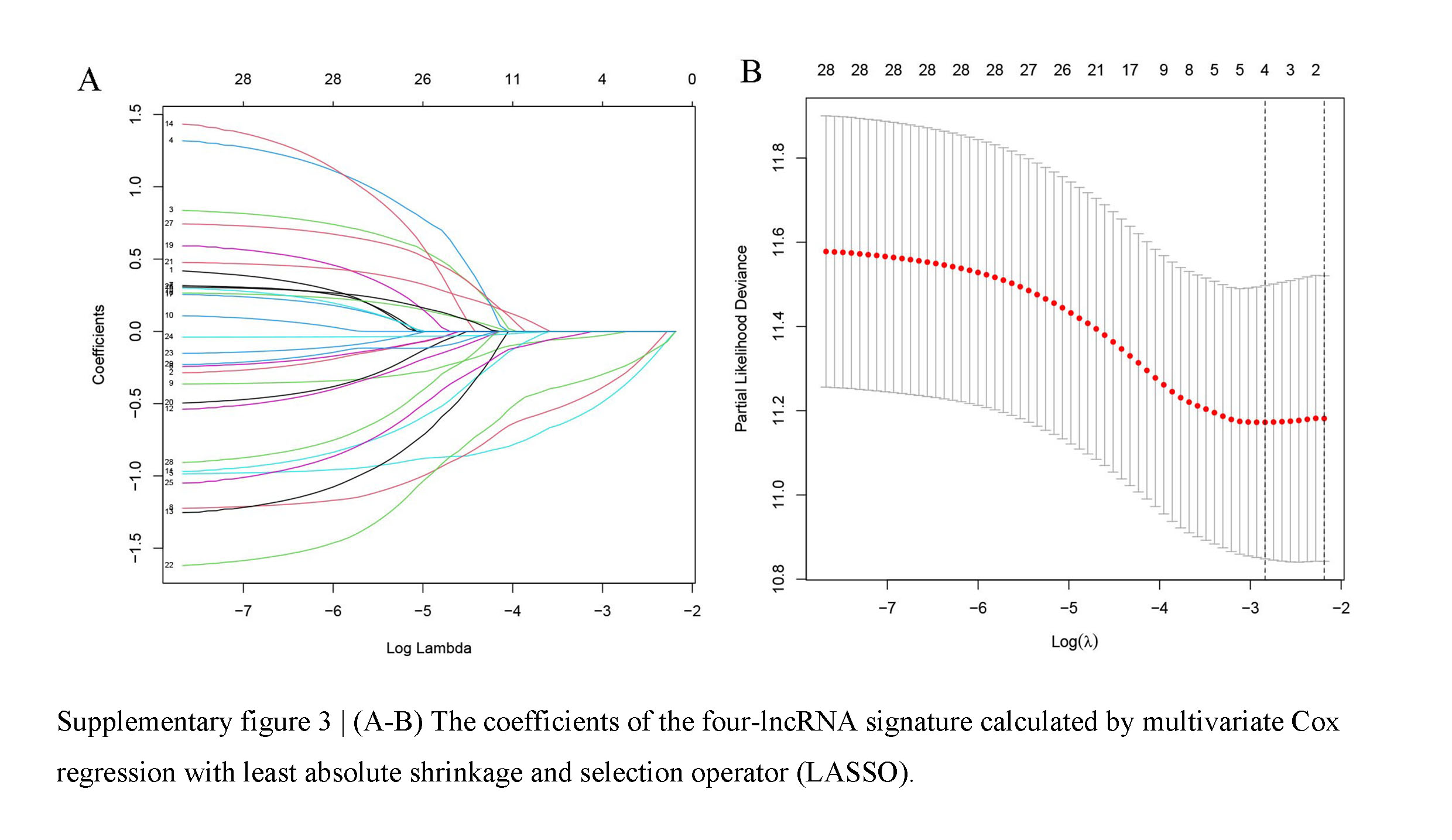

Supplement: Supplementary file 3 [file Image_3.JPEG]

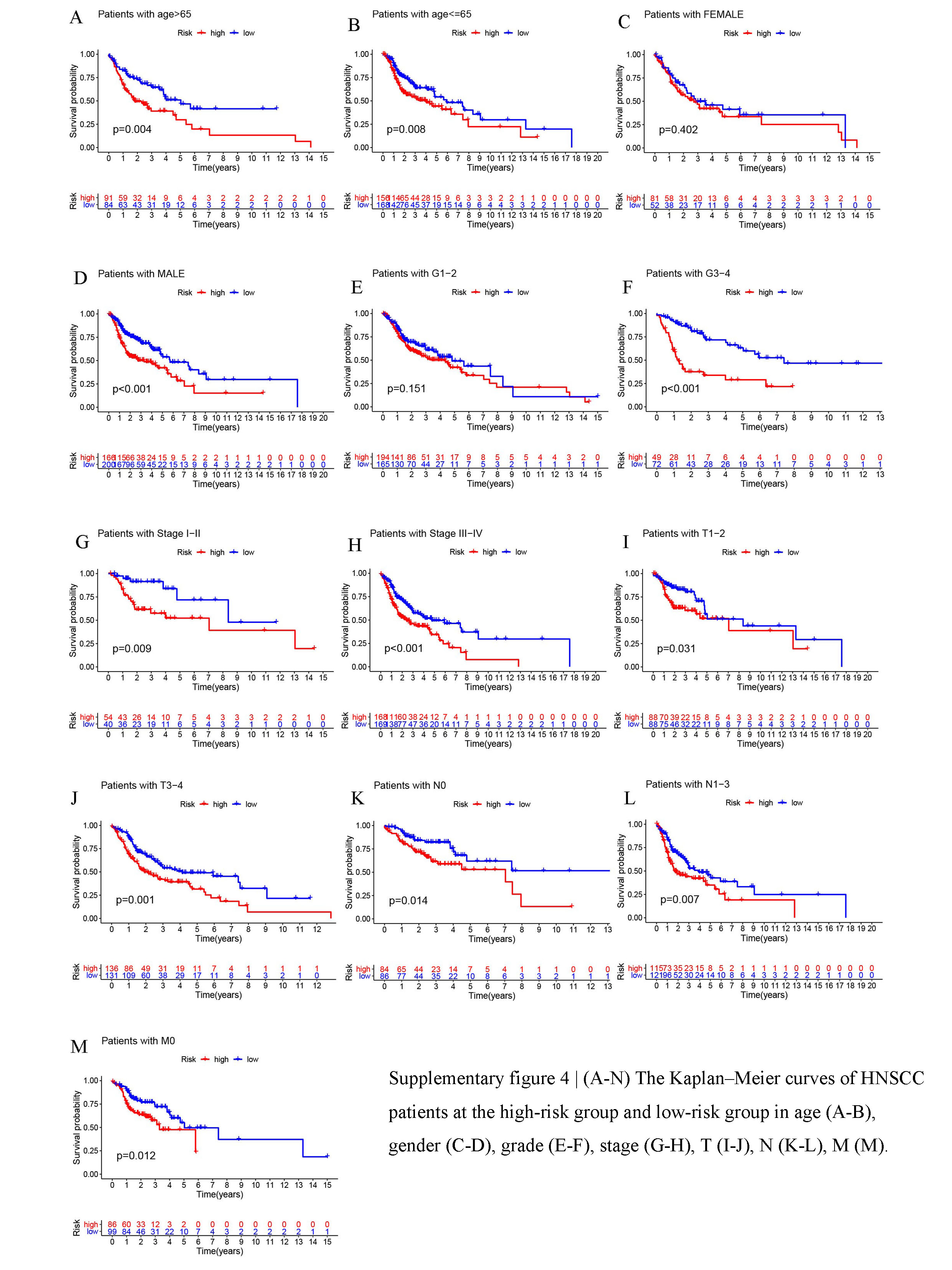

Supplement: Supplementary file 4 [file Image_4.JPEG]
